# Supplementary material for: Role and mechanism of NCAPD3 in promoting malignant behaviors in gastric cancer
Source: Front Pharmacol. 2024 Apr 22;15:1341039. doi: 10.3389/fphar.2024.1341039 (PMC11070777; doi:10.3389/fphar.2024.1341039)
Supplement: Supplementary file 11 [file DataSheet2.ZIP › GSEA/Oncogenic signature/my_analysis.Gsea.1599461962066/RAF_UP.V1_UP.html]

Details for gene set RAF\_UP.V1\_UP[GSEA]

|  || Dataset | filtered\_dataset.sample\_info.cls#WT\_versus\_NCAPD3\_MUT |
| Phenotype | sample\_info.cls#WT\_versus\_NCAPD3\_MUT |
| Upregulated in class | NCAPD3\_MUT |
| GeneSet | RAF\_UP.V1\_UP |
| Enrichment Score (ES) | -0.46856844 |
| Normalized Enrichment Score (NES) | -2.2333376 |
| Nominal p-value | 0.0026595744 |
| FDR q-value | 0.008941953 |
| FWER p-Value | 0.025 |
Table: GSEA Results Summary

  

Fig 1: Enrichment plot: RAF\_UP.V1\_UP      
 Profile of the Running ES Score & Positions of GeneSet Members on the Rank Ordered List

  

| SYMBOL | TITLE | RANK IN GENE LIST | RANK METRIC SCORE | RUNNING ES | CORE ENRICHMENT || 1 | 79789 | CLMN | 160 | 0.717 | -0.0578 | No |
| 2 | 960 | CD44 | 339 | 0.573 | -0.1401 | No |
| 3 | 57493 | HEG1 | 683 | 0.386 | -0.3563 | No |
| 4 | 5311 | PKD2 | 708 | 0.372 | -0.3437 | No |
| 5 | 25825 | BACE2 | 756 | 0.344 | -0.3500 | No |
| 6 | 8835 | SOCS2 | 840 | 0.275 | -0.3878 | No |
| 7 | 6303 | SAT1 | 850 | -0.233 | -0.3756 | No |
| 8 | 55450 | CAMK2N1 | 861 | -0.269 | -0.3612 | No |
| 9 | 6286 | S100P | 1011 | -0.412 | -0.4355 | Yes |
| 10 | 11138 | TBC1D8 | 1026 | -0.424 | -0.4116 | Yes |
| 11 | 65981 | CAPRIN2 | 1064 | -0.455 | -0.4019 | Yes |
| 12 | 9076 | CLDN1 | 1084 | -0.463 | -0.3784 | Yes |
| 13 | 7378 | UPP1 | 1111 | -0.484 | -0.3583 | Yes |
| 14 | 831 | CAST | 1160 | -0.524 | -0.3509 | Yes |
| 15 | 2119 | ETV5 | 1176 | -0.545 | -0.3180 | Yes |
| 16 | 8863 | PER3 | 1227 | -0.601 | -0.3058 | Yes |
| 17 | 2887 | GRB10 | 1259 | -0.646 | -0.2763 | Yes |
| 18 | 1956 | EGFR | 1266 | -0.657 | -0.2280 | Yes |
| 19 | 26273 | FBXO3 | 1312 | -0.723 | -0.2025 | Yes |
| 20 | 1839 | HBEGF | 1328 | -0.751 | -0.1531 | Yes |
| 21 | 26136 | TES | 1361 | -0.833 | -0.1094 | Yes |
| 22 | 4734 | NEDD4 | 1369 | -0.856 | -0.0458 | Yes |
| 23 | 64764 | CREB3L2 | 1382 | -0.930 | 0.0202 | Yes |
Table: GSEA details [plain text format]

  

Fig 2: RAF\_UP.V1\_UP      
 Blue-Pink O' Gram in the Space of the Analyzed GeneSet

  

Fig 3: RAF\_UP.V1\_UP: Random ES distribution      
 Gene set null distribution of ES for **RAF\_UP.V1\_UP**
